# Race and Ethnicity in Non-Alcoholic Fatty Liver Disease (NAFLD): A Narrative Review

**DOI:** 10.3390/nu14214556

**Published:** 2022-10-28

**Authors:** Kiarash Riazi, Mark G. Swain, Stephen E. Congly, Gilaad G. Kaplan, Abdel-Aziz Shaheen

**Affiliations:** 1 Department of Medicine, Division of Gastroenterology and Hepatology, Cumming School of Medicine, University of Calgary, Calgary, AB T2N 4Z6, Canada; 2 Department of Community Health Sciences, Cumming School of Medicine, University of Calgary, Calgary, AB T2N 4Z6, Canada; 3 O’Brien Institute for Public Health, Cumming School of Medicine, University of Calgary, Calgary, AB T2N 4Z6, Canada

**Keywords:** population groups, non-alcoholic fatty liver disease, prevalence, diet, risk factors, genetic predisposition to disease

## Abstract

Non-alcoholic fatty liver disease (NAFLD) is a significant public health concern worldwide with a complex etiology attributed to behavioural, environmental, and genetic causes. The worldwide prevalence of NAFLD is estimated to be 32.4% and constantly rising. Global data, however, indicate considerable heterogeneity among studies for both NAFLD prevalence and incidence. Identifying variables that affect the estimated epidemiological measures is essential to all stakeholders, including patients, researchers, healthcare providers, and policymakers. Besides helping with the research on disease etiology, it helps to identify individuals at risk of the disease, which in turn will outline the focus of the preventive measures and help to fittingly tailor individualized treatments, targeted prevention, screening, or treatment programs. Several studies suggest differences in the prevalence and severity of NAFLD by race or ethnicity, which may be linked to differences in lifestyle, diet, metabolic comorbidity profile, and genetic background, among others. Race/ethnicity research is essential as it can provide valuable information regarding biological and genetic differences among people with similar cultural, dietary, and geographical backgrounds. In this review, we examined the existing literature on race/ethnicity differences in susceptibility to NAFLD and discussed the contributing variables to such differences, including diet and physical activity, the comorbidity profile, and genetic susceptibility. We also reviewed the limitations of race/ethnicity studies in NAFLD.

## 1. Introduction

Non-alcoholic fatty liver disease (NAFLD) is a significant public health concern in many countries, especially in the western world [1,2]. NAFLD is not widely recognized as a public health issue, even though it is directly associated with metabolic syndrome (MetS), a cluster of conditions associated with insulin resistance, including abdominal obesity, hypertension, hyperlipidemia (high triglyceride and low HDL levels), and diabetes (impaired glucose tolerance test, or overt type 2 diabetes mellitus (T2DM)) [3,4]. NAFLD is one of the leading causes of chronic liver disease. An increased prevalence of NAFLD and consequently of NASH (non-alcoholic steatohepatitis, an advanced form of NAFLD) could result in a rise in the cases of cirrhosis and hepatocellular carcinoma (HCC), thus adding to the burden of disease and the overall cost of health care [1,2,5]. The most effective treatment strategies against NAFLD remain preventive measures through regular physical activity, low-calorie eating, and weight loss [1]. A critical public health concern with NAFLD is that the disease remains undiagnosed in most patients [6]. When it advances to NASH, for which there is no proven medical therapy, the prognosis worsens with an increased risk of developing end-stage liver disease and HCC [5,7]. Furthermore, the co-existence of metabolic comorbidities like T2DM increases the risk of NAFLD progression to NASH [8]. Therefore, the early screening and diagnosis of NAFLD, besides modifying its metabolic risk factors, are essential to reversing the progression of the disease [9].

The epidemiology and demographic characteristics of NAFLD vary from place to place. Our recent report estimated that the overall prevalence of NAFLD worldwide has been 32.4%, with a steady rise from 25.5% according to the reports of 2005 or before to 37.8% from 2016 or later [10]. The worldwide incidence of NAFLD was estimated to be 46.9 cases per 1000 person-years [10]. An important finding of our study was that there was considerable heterogeneity among studies for both NAFLD prevalence (*I*^2^ = 99.9%) and NAFLD incidence (*I*^2^ = 99.9%) [10], which indicates differences in the outcomes between the included studies [11]. Exploring the heterogeneity is vital because it could limit the generalizability of research findings [12,13,14,15]. Identifying variables that affect the estimated prevalence or incidence of a disease (i.e., effect-measure modifiers, like demographic variables such as age, sex, income, comorbidities, or other disease risk factors) is essential to all stakeholders, including patients, researchers, health care providers, and policymakers. Detecting these variables not only helps with the research on disease etiology but also aids in recognizing who is possibly more or less at risk of the disease, which in turn will define the focus of the preventive measures and help appropriately tailor individualized treatments [13]. The risk of NAFLD might not be equal among every individual in the general population. For example, the young, the old, females, and people with diabetes have specific susceptibility to the disease [13].

Racial and ethnic differences in various health outcomes regularly appear in medical literature, implying a role for racial and ethnic characteristics in the pathophysiology of the disease and in determining disease risk [16,17,18]. NAFLD has a complex etiology stemming from an interaction between modifiable and non-modifiable variables, including genetic, behavioural, and environmental factors [19,20]. Several studies suggest differences in the prevalence and severity of NAFLD by race or ethnicity [21,22,23,24,25]. Nonetheless, the comparative epidemiologic research on NAFLD prevalence among different ethnicities is not substantial and is currently limited to only a few countries, especially the U.S. Race/ethnicity research is important, particularly for diverse countries like the U.S., as such epidemiologic data provide information on biological and pathophysiological and risk factors differences regarding the disease among ethnicities. This, in turn, helps understand the disease etiology, the interplay between genetic and environmental factors, as well as the generalizability of global data in tailoring public health measures focused on targeted prevention, screening, or treatment programs [26,27].

In this publication, we first review the existing literature on the race/ethnicity differences in susceptibility to NAFLD and discuss lifestyle (diet and physical activity), the comorbidity profile, and genetic susceptibility as contributing variables to such differences. We then discuss the issues related to the study of race/ethnicity in NAFLD and the obstacles to the generalizability of such findings.

## 2. Racial/Ethnic Differences in Susceptibility to NAFLD

The term “race” is historically aimed at describing biological inheritance, while “ethnicity” refers to specific groups with shared social and cultural traditions, geographic origins, ancestry, family patterns, and some biological attributes [26,28,29]. In the United States, most studies follow the race and ethnicity guidelines provided by the U.S. Office of Management and Budget (OMB) and are used by the U.S. Census Bureau for data collection [30,31]. However, the questionnaire provided to the participants does not reflect any biological or anthropological definition of race and attempts to define it as primarily a social construct, i.e., ethnicity [31]. The data relating to race, defined as a person’s self-identification with one or more social groups, is collected for five major groups: White or Caucasian, Black or African American, Asian, American Indian, or Alaska Native, and Native Hawaiian or Other Pacific Islander. On the other hand, ethnicity in the U.S. refers to whether an individual self-identifies as Hispanic/Latino or not, which may be based on the lineage, nationality, or geographical origin of individuals (or their parents) before arriving in the United States [30,31]. Race information is required for many Federal programs and is critical in making policy decisions, particularly for civil rights. States use these data to meet legislative redistricting principles. Race/ethnicity data also are used to promote equal employment opportunities and to assess racial disparities in health and environmental risks [30,31].

The number of published race/ethnicity studies from the U.S. is sizeable; nevertheless, many focus on the patient cohorts from the National Health and Nutrition Examination Survey (NHANES), a cross-sectional study conducted by the National Center for Health Statistics. The participants in NHANES are recruited as a nationally representative sample of about 5000 people each year living in counties across the United States [21]. The earliest study of race/ethnicity differences in NAFLD prevalence is from the third NHANES from 1988 to 1994. While the third NHANES was not initially designed to assess NAFLD, a retrospective reassessment of the available gall bladder ultrasound records for a subset of the study population yielded a prevalence estimate for fatty liver [23]. A report by Huang et al., 2021, based on the third NHANES has shown that the Hispanic population had a higher prevalence of NAFLD at 37.0%, and the non-Hispanic Black population had a lower prevalence of NAFLD at 24.7%, compared to the non-Hispanic White population with 29.3% [22]. More comprehensive research was performed by Rich et al., 2018 [24], by conducting a systematic review and meta-analysis of published reports up to 2016. They estimated the pooled relative risk (R.R.) for NAFLD, which is calculated by combining the ratios of the probabilities of NAFLD occurring in a race group versus the probability of NAFLD occurring in another race group from different papers using meta-analysis methods [32]. They showed that in population-based studies performed in the U.S., NAFLD was higher in the Hispanic population (22.9% (95% CI: 21.6–24.1%)) and lower in the Black population (13.0% (95% CI: 12.2–13.9%)) compared to the non-Hispanic White population (14.4% (95% CI: 14.0–14.8%)) [24]. These findings would translate into a pooled R.R. of 1.47 (95% CI: 1.35–1.61) for NAFLD in the Hispanic population and 0.74 (95% CI: 0.69–0.80) in the Black population when compared to White populations [24]. In high-risk patients (i.e., in the presence of obesity, diabetes, or a history of chronic liver disease), the Black population still had a lower risk of NAFLD than the White population (pooled R.R.: 0.85 (95% CI: 0.75–0.97)) [24]. The comparison, however, was not straightforward for Hispanic populations versus White populations and only became borderline positive after eliminating the outlier studies (1.16 (95% CI: 1.03–1.33)). The prevalence of NASH (a representation of NAFLD severity) was higher in Hispanic individuals (pooled R.R. = 1.24 (95% CI: 1.02–1.52)) and lower in the Black population (pooled R.R. = 0.72 (95% CI: 0.60–0.87)) compared to the White population. Nevertheless, neither the Hispanic nor Black populations were significantly different from the White population in terms of the stage of fibrosis (measured by the pooled proportion of NAFLD patients with significant stage F3–F4 fibrosis [24] (liver fibrosis histological staging: no fibrosis = F0, mild fibrosis = F1, moderate fibrosis = F2, severe fibrosis = F3, and Cirrhosis = F4) [33]). Consistent with the previous reports, a recent study on the 2017–2018 phase of the NHANES study assessed fatty liver with the controlled attenuation parameter (CAP), a sensitive diagnostic measure for fatty liver. This study showed a higher NAFLD prevalence in Hispanic individuals (63.7%) and a lower prevalence in Black individuals (46.2%) compared to White individuals (56.8%) [34]. However, the prevalence of significant NAFLD fibrosis (F2–F4) by Fibroscan^®^ (Echosens, Paris, France), a special ultrasound technology capable of measuring liver stiffness and fatty changes [35], was not statistically different among the Hispanic population (15.4% (95% CI: 10.4–20.5%), compared to non-Hispanic White individuals (14.6% (95% CI: 10.8–18.5)) or non-Hispanic Black individuals (14.6 (95% CI: 11.2–18.0)).

Outside the U.S., the studies of NAFLD prevalence stratified by race/ethnicity are limited. Zhou et al., 2019, in their systematic review and meta-analysis of the nationwide prevalence of NAFLD in China, demonstrated a significantly higher prevalence of NAFLD among Uyghur ethnics compared to Han ethnics, Kazakhs, or Mongolians (46.6% vs. 29.3%, 24.3%, and 25.0%, respectively) [25]. A recent multiethnic, population-based prospective study by de Groot and colleagues (2022) [36] on children aged 8–10 from the largest ethnic minorities in the Netherlands has shown that, compared to children with a Dutch background, the children with Surinamese-Creole, Cape Verdean, Moroccan, Dutch Antillean, or Turkish backgrounds had a higher total liver fat fraction measured using MRI. Among them, the children with a Turkish background had the highest median liver fat percentage (2.5% (95% CI: 1.2–10.7%)) and NAFLD prevalence (9.1%) [36]. The higher liver fat fraction observed in children with a Turkish background persisted even after controlling for body mass index (BMI) and several other maternal or perinatal factors, including maternal age, maternal education, maternal pre-pregnancy BMI, smoking during pregnancy, alcohol use during pregnancy, and birth weight, breastfeeding, sugar intake at infancy, screen time, and exercise. Since pediatric NAFLD tends to persist into adulthood [37], a propensity to have a higher liver fat fraction from childhood among Turkish ethnics could partly explain why Turkey has an adult NAFLD prevalence as high as 48.4%, the highest among European nations [10,37].

**Figure 1:**
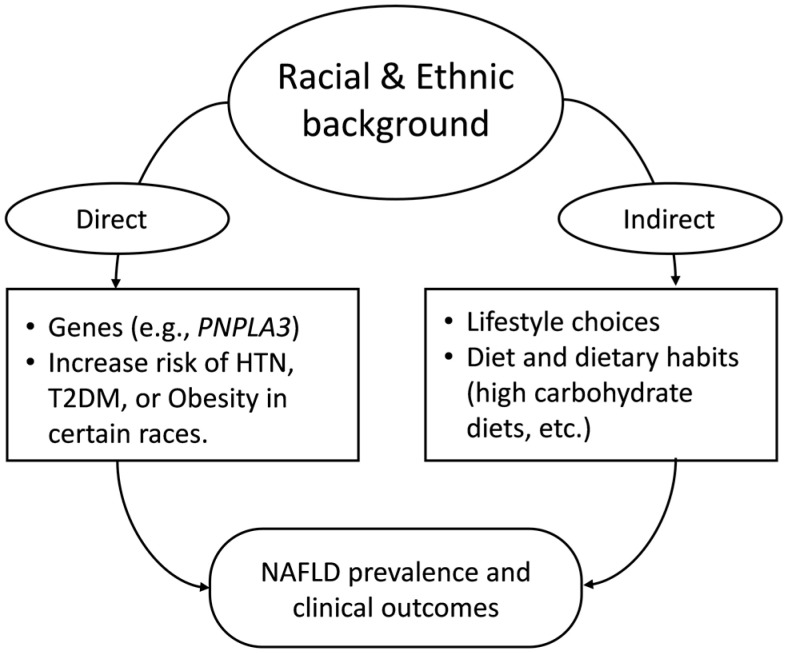
Simple representation of the topics discussed in this review. *PNPLA3* (Patatin-like phospholipase domain-containing protein 3), HTN (Hypertension), T2DM (Type 2 Diabetes Mellitus), NAFLD (Non-alcoholic Fatty Liver Disease).

Although limited, the existing reports from around the world indicate differences in susceptibility to NAFLD among races/ethnicities. Many have attributed the observed racial/ethnic differences in NAFLD susceptibility to variables including lifestyle, dietary habits, comorbidity profiles, genetic factors, socioeconomic status, etc., or a combination of multiple such factors [38,39]. Figure 1 is a simple representation of the topics discussed in this review.

## 3. Role of Diet and Physical Activity

People follow dietary patterns influenced by their cultural and ethnic backgrounds. A dietary pattern is defined as the proportions, diversities, or combinations of various foods and drinks in a diet and the frequency with which they are routinely consumed [40]. Ethnic foods, however, are influenced by several variables, including social (socioeconomic status, religion, education, etc.) and geographical factors (availability of food and raw ingredients, local farming practices, supply of industrially or naturally prepared foods, etc.) [41,42]. Several dietary patterns have been characterized, including the Mediterranean, Western, and Prudent dietary patterns, to name a few [41].

Long-term adherence to a low-calorie diet decreases liver fat [1,43]. Reducing caloric intake by at least 30% (equal to roughly 750–1000 kcal/day) improves NAFLD and insulin resistance [43]. Dietary patterns which are low in high glycemic index foods and saturated fats but high in dietary fibre, monounsaturated fatty acid, and polyunsaturated fatty acid intake have a positive effect on gut microbiota, intestinal barrier function, and intrahepatic triglyceride (IHTG) accumulation [44]. Such dietary patterns can reduce the development of NASH, possibly without any perceivable weight loss [1]. Several dietary elements and patterns have been associated with either promoting or preventing NAFLD [1]. The association between dietary patterns with NAFLD has already been discussed in our previous review [1]. The Western dietary pattern emerged after the introduction of new methods of food processing during the Industrial Revolution. This dietary pattern includes high amounts of red meat, snacks, sweets, high amounts of saturated fats, fructose, cholesterol-rich sources, and sugar-packed soft drinks, and low in dietary fibres, fruits, and vegetables [1,41,45]. Western dietary pattern predisposes individuals to NAFLD directly or indirectly by contributing to weight gain [1,41]. A recent systematic review and meta-analysis by Hassani Zadeh et al., 2021, which aimed at evaluating the association of dietary patterns and NAFLD, has demonstrated that the Western dietary pattern was significantly associated with a higher prevalence of NAFLD (Odds Ratio (OR) = 1.56 (95% CI: 1.27–1.92)) [46]. In contrast, a higher adherence to a prudent dietary pattern, which consists of fruit, vegetables, salad, unprocessed grains, lean meat, and fish [47], significantly reduced the risk of NAFLD (OR = 0.78 (95% CI: 0.71–0.85)) [46]. The Mediterranean diet, another dietary pattern rich in fruits, vegetables, whole grains, nuts, legumes, and fish but low in red and processed meat, high-in-sugar foods, and refined carbohydrates, also improves NAFLD [1,48,49,50].

Dietary differences have been linked to ethnic disparities associated with NAFLD [51,52]. Differences in exercise and dietary behaviours exist in various ethnic groups [51,53]. However, the association of the NAFLD risk in ethnicity with their dietary habits is not yet well supported. It is, however, known that some aspects of diet and weight control would predispose specific populations to NAFLD [1,54]. For example, the Mediterranean diet, inspired by the eating habits of olive-producing Mediterranean countries before the 60s, specifically Greece, Italy, Spain, and France [55], has been linked with a lower prevalence of NAFLD or even has been recommended as a preventive solution for NAFLD [1]. Nevertheless, several Mediterranean countries currently have some of the highest NAFLD prevalence estimates in the world, including Greece at 41% [56] and Italy at 38.2% [10]. Such observations might indicate that the dietary patterns of the population groups of the region have probably changed over the past few decades. Some authors associate such discrepancies with the dissemination and increased popularity of the Western-type dietary pattern worldwide [51,57]. A recent report by Martimianaki et al., 2022 [58], signifies that only 28.3% of the Greek adult population have high adherence to the Greek traditional Mediterranean diet, most of which results from higher adherence (39.7%) by participants of 65 years or older. Younger individuals were more adherent to a Western diet pattern characterized by the consumption of red meat, animal fats, and cheese, with only 25.5% adherence to the Mediterranean diet. Kalafati et al., 2018 [59], studied the association of dietary patterns with NAFLD in a sample population from Greece. They recognized four dietary patterns, including the fast food-type pattern, prudent pattern, high-protein pattern, and unsaturated fatty acids pattern. They showed that the fast food-type pattern (which included most of the main components of the Western dietary pattern) was both associated with significantly higher odds of NAFLD and with higher levels of C-reactive protein and uric acid (two NAFLD-related biomarkers) [59]. In contrast, the unsaturated fatty acids pattern (rich in nuts, chocolate, and other foods rich in monounsaturated and polyunsaturated fatty acids) was negatively associated with NAFLD, lower insulin levels, and the homeostatic model assessment of insulin resistance [59]. While the prudent pattern was negatively associated with triglyceride and uric acid levels, the high-protein pattern was not associated with any NAFLD-related biomarker [59]. The shift from the dietary habits of several other ethnic groups living in other parts of Europe toward the Western dietary pattern, especially among younger individuals, has been demonstrated and attributed to the acculturation and adoption of a Western lifestyle [60].

India is a multiethnic nation with rich and highly varied cuisines. Furthermore, people from India are at higher risk for metabolic diseases like T2DM than White individuals, and they have a relatively high national NAFLD prevalence of 25.7% [10,61]. A recent case–control study by Vijay et al., 2022, performed on the Trivandrum NAFLD cohort attempted to investigate the association of individuals’ dietary intake and composition of the local recipes with clinical outcomes regarding NAFLD [62]. Trivandrum, the southernmost district in Kerala, India, is an area with NAFLD prevalence reports as high as 51% [63]. The population-based Trivandrum NAFLD cohort has been designed in the past decade to examine the interaction between lifestyle factors and genetics and the risk of NAFLD [62,63]. They assessed NAFLD using ultrasound, followed by the assessment of liver fibrosis in NAFLD patients using transient elastography (T.E.) via Fibroscan^®^ (Echosens, Paris, France). They demonstrated that NAFLD cases, compared with controls, consumed significantly higher amounts of refined rice, red meat, and refined sugars but lower amounts of vegetables, legumes, milk products, nuts, and oil seeds. Furthermore, consuming fried and roasted foods was positively and boiled, steamed, and uncooked/unprocessed foods were negatively associated with NAFLD status (OR = 1.49 (95% CI: 1.22–1.78) vs. OR = 0.43 (95% CI: 0.20–0.48), respectively). Significant fibrosis was positively associated with higher consumption of fats, edible oils, red meat, and fried foods, while negatively associated with the consumption of leafy vegetables [62]. They linked the association of frying food with NAFLD to the possible oxidation of fats due to heat exposure, the formation of toxic compounds such as radical species, and subsequent liver cell damage [62].

Japan has a low national NAFLD prevalence of 22.3% [10]. A low prevalence of NAFLD in the Japanese population has been associated with a dietary pattern rich in vitamins, fibre, iron, and potassium [54]. However, rice is also the primary food staple in many Asian countries, including Japan [64]. A study from Japan by Tajima et al., 2017 [65], examined the impact of the consumption of carbohydrate-rich foods (rice, bread, and noodles) and the percentage of energy intake from carbohydrates on the prevalence of NAFLD in Japanese middle-aged men and women. Rice was the major contributor to dietary carbohydrate intake (41.0% in men and 34.5% in women), followed by bread (8.8% in men and 9.7% in women) and noodles (9.2% in men and 7.0% in women). In women, carbohydrate intake was positively associated with the prevalence of NAFLD. However, in men, the association was not statistically significant in the multivariable-adjusted model. Rice intake was also positively associated with the prevalence of NAFLD in women (the fully adjusted OR for the highest compared with the lowest quartile = 1.87 (95% CI: 1.03–3.41)). In both sexes, there was no association between bread intake and the prevalence of NAFLD. Their findings suggested that dietary carbohydrates and rice (the primary dietary carbohydrate source of participants in Japanese) might be strongly associated with NAFLD, mainly in Japanese women. Their finding is supported by a previous study which showed a similar association between increased intakes of white rice and the increased risk of T2DM among Japanese women but not men [66]. Comparing the research results from the two papers from India and Japan would suggest that the association of staple foods with NAFLD can be influenced by the intrinsic biological characteristics between different races/ethnicities.

The traditional Chinese dietary pattern, while high in carbohydrates, is vegetable-rich and believed to be low-risk for NAFLD [67]. Yet, in China, NAFLD is a seriously surging health burden [25], affecting 32.5% of the general adult population in China [10]. Zhang et al., 2021 [68], investigated the association between dietary patterns and the risk of incident NAFLD in a large sample of the general Chinese adult population (*n* = 17,360), followed up for an average of 4.2 years. Their study recognized three major dietary patterns among the study population. They included a sugar-rich dietary pattern (involving a high intake of kiwi fruit, strawberry, persimmon, candied fruits, sweets, and Chinese cakes), a vegetable-rich dietary pattern (high consumption of green leafy vegetables, Chinese cabbage, cucumber, celery, and pumpkin), and an animal food dietary pattern (characterized by high consumption of animal products like organs, blood, sausages, and pork skin, as well as preserved eggs and instant noodles). They showed that, in Chinese adults, while following a vegetable-rich dietary pattern was not associated with a higher risk of NAFLD, adherence to animal food or sugar-rich dietary patterns increased the risk of NAFLD significantly [68]. The authors associated the lack of protective effect for vegetable-rich dietary patterns against NAFLD, with the practice of heavily cooking most of the consumed vegetables in Chinese culture [68].

Overall, the current literature on the different dietary patterns of various geographical regions and ethnicities points out some similarities between the association of diet and susceptibility to NAFLD. While people with different ethnic backgrounds might follow different dietary patterns, they all have recognizable unhealthy dietary and food preparation habits. Adherence to diets rich in carbohydrates (white rice, sugar-rich fruits, snacks, or soft drinks) or cholesterol-rich foods like red and processed meats and low in fibre, fruits, and vegetables is unanimously shown to be associated with NAFLD. Moreover, the Western dietary pattern is now a global crisis and is increasingly recognized as a significant variable in the pathophysiology of NAFLD.

Remarkable research by Noureddin et al., 2020, on a diverse ethnic population in the U.S. showed that diets high in cholesterol-rich foods like red and processed meats and low in fibre were associated with NAFLD, independent of race and ethnic groups [69]. This might indicate that the more substantial effect of lifestyle changes and environmental factors resulting in unhealthy dietary habits and a lack of physical activity might conceal the more subtle racial/ethnic differences in NAFLD susceptibility. As already implicated in the obesity epidemic, several environmental factors promote the rise of metabolic diseases, including obesity and NAFLD, by encouraging energy consumption and discouraging energy expenditure [70,71]. The two main features of a Western lifestyle include Western dietary patterns and sedentary behaviour. As mentioned earlier, the Western diet mainly comprises large portions of readily available, good-tasting, inexpensive, and energy-dense foods [70,71]. Sedentary behaviour depicts a reduction in energy expenditure in the form of jobs with decreased physical labour, easy commutes, decreased physical activity at school and in daily living, and increased time spent on sitting activities such as watching television and Web surfing [70,71]. Sedentary behaviour has been increasingly accepted as an independent risk factor for NAFLD [72], and physical activity like aerobic exercise can protect against NAFLD by decreasing body fat [51]. In patients with NAFLD, increasing physical activity improves insulin resistance, decreases intrahepatic triglyceride content, and reduces the markers of hepatocellular injury independent of weight loss [73,74]. Such benefits are even more prominent when weight loss occurs [75]. The systematic reviews of the association of diet and exercise with NAFLD prevalence are limited. Zhou et al., 2019, showed that in China, a lifestyle that integrates exercise significantly decreased the odds of NAFLD (OR = 0.78 (95% CI: 0.68–0.89)) based on the results of 10 publications [25]. The impact of such environmental factors on NAFLD risk, as well as possible solutions for lifestyle modifications and environmental policies, to revert possible impacts and prevention of NAFLD needs extensive research.

Assuming rural communities are less affected by the Western-type dietary changes than urban communities, one could hypothesize that the prevalence of NAFLD is lower in rural than urban areas. While our recent study showed a trend toward lower NAFLD prevalence in rural areas compared with urban settings (23.7% (95% CI: 15.5–31.8) vs. 32.4% (95% CI: 28.6–36.1)), the difference did not reach statistical significance [10]. Comparably, the study by Li et al., 2019 [76], did not show a significant difference either. However, this might be due to the low number of rural prevalence reports available in both studies (*n* = 7 in our study and *n* = 3 in the study by Li et al.) [10,76].

## 4. Role of Metabolic Comorbidities

MetS and its components (i.e., abdominal obesity, hypertension, hyperlipidemia, and diabetes) are well-known comorbidities associated with NAFLD [77,78]. In a study on a group of healthy Korean adults, Lee, 2010, reported that the MetS at the baseline increases the risk of developing NAFLD by almost six times [79], and even one or two components of the MetS at the baseline make the risk 2.4-fold [79]. The prevalence of T2DM is increasing globally and correlates with the increased prevalence of NAFLD [80]. It is reported that the prevalence of NAFLD/NASH in patients with T2DM ranges between 50 to 75% and depends on the patients’ ethnicities [81,82]. T2DM [8] and hypertension [83,84,85] are not only risk factors for the development of NAFLD but also accelerate the progression of NAFLD to advanced liver disease and increase the mortality risk. Similar associations exist between dyslipidemias, including hypercholesterolemia and hypertriglyceridemia with NAFLD [83,84,86].

The strong association of NAFLD with obesity, an important component of MetS, is well known. Obesity is the most significant modifiable risk factor for NAFLD [1]. While NAFLD prevalence is around 30% in the general population, it climbs to 50-90% in obese subjects [87]. Both obesity and NAFLD are associated with over-nutrition, a Western-type diet, and a sedentary lifestyle [1,87]. NAFLD prevalence correlates with the prevalence and degree of obesity [1,87]. NAFLD has a prevalence of 65 and 85% in patients with grade I–II obesity (BMI = 30–39.9 kg/m^2^) and grade III obesity (BMI = 40–59 kg/m^2^), respectively [87]. Because of such associations between MetS and its components with NAFLD, the metabolic comorbidity profile could result in considerable diversity in susceptibility to NAFLD among individuals.

As discussed earlier, compared to the non-Hispanic White population, the prevalence of NAFLD is lower in the non-Hispanic Black population and higher in the Hispanic population in the U.S. The prevalence of MetS, however, does not follow the same pattern among the three major groups. Moore et al., 2017 [88] calculated the odds of NAFLD after adjustment for education, poverty-to-income ratio, and age for the 2007–2012 periods of NHANES and showed that, while for females, there were no differences in the odds of NAFLD between the Mexican American or Black populations compared to the White population (adjusted odds ratio (aOR) = 1.30 (0.97–1.75) and 1.06 (0.82–1.35), respectively), among males, only the Black population showed significantly lower odds compared to the White population but not the Mexican American population (aOR = 0.74 (0.62–0.89), and 0.91 (0.70–1.19), respectively). A recent study on the 2017–2018 cohort of NHANES by Zhou et al., 2022 [89], investigated the association of metabolic syndrome components with NAFLD measured by the Controlled Attenuated Parameter (CAP). While obesity (quantified as a 10 cm increase in waist circumference) was positively associated with NAFLD in all race groups (i.e., White, Asian, Hispanic, and Black), T2DM was associated with NAFLD in the White, Asian, and Hispanic groups, but not in the Black population. Hypertension was associated with NAFLD only in White individuals and Asian individuals but neither in the Black nor Hispanic population. Conversely, hyperlipidemia was only associated with NAFLD in the Black population. Additionally, significant fibrosis, an indication of the severity of NAFLD, was associated with T2DM in all groups except for the Black population [89]. The observation indicates that the associations of NAFLD with different components of MetS are not identical among different race groups.

Browning et al., 2004 [90], examined the association between hepatic triglyceride content (HTGC) and body fat distribution among the African American, Hispanic, and Caucasian participants of the Dallas Heart Study. The Dallas Heart Study was a multiethnic, population-based study on 18–65-year-old residents of Dallas County, Texas, which was designed to examine the ethnic differences in cardiovascular risk [90]. They showed that the median HTGC for Hispanic individuals (4.6%) was significantly higher than for White (3.6%) or Black individuals (3.2%). All three groups significantly correlated with the Homeostatic Model Assessment of Insulin Resistance values (HOMA IR, a test for the assessment of insulin resistance) as well as the components of the MetS, including BMI and waist circumference [90]. The correlation coefficient for BMI and HTGC was highest in the Hispanic population and lowest in the Black population [90]. For Hepatic steatosis, however, the higher prevalence of hepatic steatosis (HTGC > 5.5%) was positively correlated with obesity and insulin resistance in the Hispanic population. In contrast, the lower prevalence of hepatic steatosis in Black individuals was not attributable to insulin resistance [90]. An interesting finding in their study was that the median HTGC showed sex-dependence among White individuals, significantly higher in males than females (4.4%, vs. 3.0%), but not in the Hispanic or Black populations; that also stayed true when the prevalence of NAFLD was compared (42% vs. 24%), indicating that the sex differences in NAFLD could be affected by ethnicity. In Black individuals, despite a similar prevalence of obesity and insulin resistance to the Hispanic population, the prevalence of NAFLD was significantly lower (24% vs. 45%). They concluded that in the Hispanic population, unlike the Black population, a high prevalence of NAFLD might be attributable to a higher prevalence of obesity and insulin resistance. Black individuals also had lower plasma HDL levels and a lower prevalence of hypertriglyceridemia, which may indicate profound differences in lipid homeostasis between the studied ethnicities. In a follow-up to the same study, Guerrero et al., 2009, determined that ethnic differences in the levels of HTGC and prevalence of NAFLD were not mediated by differences in overall obesity but could partly be associated with the propensity to accumulate intraperitoneal fat [91]. They demonstrated that intraperitoneal fat was directly associated with the severity of HTGC, irrespective of ethnicity. On the other hand, African Americans tended to have proportionally less intraperitoneal fat than Caucasian or Hispanic individuals [91]. Despite adjustment for total adiposity, the differences in HTGC observed between the ethnicities persisted [91]. To support that finding, diabetes and overall obesity are more common among Black than White individuals. However, the latter group has a significantly higher risk of NAFLD [92,93,94], suggesting that the factors accountable for reduced intraperitoneal fat deposition in Black individuals might be causally linked to the reduced propensity for this group to develop NAFLD. The low propensity for intraperitoneal fat deposition in Black individuals despite increased overall obesity, however, does not protect them against insulin resistance, which can explain the absence of many of the disorders in lipid metabolism typically associated with insulin resistance in African Americans [91].

Li et al. [95] also found a positive association between visceral adiposity index (VAI), an indicator of intraperitoneal fat deposition, and NAFLD in the Hispanic and non-Hispanic White populations but not in the non-Hispanic Black population. In another study, in a multiethnic cohort from Hawaii and Los Angeles, Lim et al., 2019, compared the measured trunk, visceral, and liver fat among different ethnic/racial groups, using whole-body dual-energy X-ray absorptiometry (DXA) scans and magnetic resonance imaging (MRI) to study if fat storage was associated with racial/ethnic disparities in the MetS and NAFLD [96]. Trunk, visceral, and liver fat were highest for Japanese Americans, intermediate for Native Hawaiians, Latinos, and White individuals, and lowest for African Americans, with an even stronger association after adjusting for total fat mass. After adjustment for age, height, and total fat, Japanese Americans had the highest prevalence of NAFLD, while African Americans had the lowest. The association of weight gain with both intraperitoneal fat and liver fat was significantly different by ethnicity and was again greatest in Japanese Americans and lowest in African Americans [96]. They concluded that the propensity to store fat intraperitoneally was associated with the risk of MetS and NAFLD, and a low propensity could partly be a protective factor in Black individuals [96].

Overall, the current literature indicates phenotypic differences in NAFLD pathophysiology among ethnicities, especially when comparing the Black population with other ethnicities. Black individuals are found to be more resistant to developing NAFLD compared to other races. Ethnic/racial differences in the tendency to store fat intraperitoneally are possibly a major explanatory factor, not only to ethnic/racial differences in NAFLD development but also as a risk factor for NAFLD in general.

## 5. Genetic Susceptibility to NAFLD

Besides metabolic and environmental factors, extensive research in the last decade has identified many genetic changes that may be associated with the development of NAFLD and NASH [19,97]. There is ample epidemiological evidence on the role of genetic susceptibility in NAFLD, mainly from studies showing the clustering of NAFLD cases among families and observing differences in the prevalence and severity of NAFLD among ethnicities [98]. Genetic susceptibility variations may explain a significant portion of heterogeneity observed in the NAFLD prevalence/incidence estimates from different geographical regions [10] and the variability in disease severity among individuals. Genetic factors have been suggested as one of the underlying causes of racial/ethnic differences in susceptibility to NAFLD [99]. The four genes most reported to be associated with NAFLD include Patatin-like phospholipase domain-containing protein 3 (*PNPLA3)*, Transmembrane 6 superfamily 2 (*TM6SF2)*, Membrane-Bound O-acyltransferase Domain Containing 7 (*MBOAT7)*, and glucokinase regulator (*GCKR)* [99].

Patatin-like phospholipase domain-containing protein 3 (*PNPLA3*) is a lipid droplet-associated protein that hydrolases triglycerides and retinyl esters [100] in adipocytes and hepatocytes, in which it helps regulate both lipogenesis and lipolysis [101,102]. The expression of the *PNPLA3* gene decreases during fasting and increases after eating, suggesting a role for *PNPLA3* protein in processing and storing fats in the diet [103]. A variant of the *PNPLA3* gene is rs738409, which is a single nucleotide substitution of cytosine (C allele) to guanine (G allele), resulting in the replacement of isoleucine with methionine at position 148 (I148M) of the protein [104]. This nonsynonymous change in encoding results in a loss of function in the *PNPLA3* protein, leading to an increased accumulation of triglycerides in lipid droplets within hepatocytes [105]. It has been shown that the G allele (either heterozygous or homozygous) is not only associated with NAFLD development but also with more advanced liver fibrosis and NASH [102,106]. This gene variant is believed to be the most important genetic determinant of the full spectrum of NAFLD, from development to progression to NASH and HCC [107]. The frequency of this variant is about 30% of the whole cohort in the Multiethnic Cohort Study of Diet and Cancer (MEC) [108]. The variant was associated with an almost 40% increase in the risk of NAFLD among the cohort population (OR = 1.39 (95% CI: 1.28–1.52)) [108]. The MEC cohort was established in Hawaii and Los Angeles in the mid-90s to help study the association between diet and other lifestyle factors and cancer [109]. The *PNPLA3* rs738409 variant has been implicated in various ethnicities, including the Hispanic, African American, East Asian, and South Asian populations [51]. The *PNPLA3* rs738409 variant is primarily associated with a lean NAFLD phenotype from Asia [51]. Hispanic individuals show a high frequency of the rs738409 variant compared to the total cohort (46% vs. 33%), and the variant is associated with a 46% increase in the risk of NAFLD (OR = 1.54 (95% CI: 1.27–1.88)) [108]. African Americans, on the other side, show a lower frequency of the G allele (15%), but the allele is associated with a 46% increase in the risk of NAFLD (OR = 1.46 (95% CI: 0.93–2.21)) [108]. According to the study by Bonacini et al., 2021, in the presence of GG alleles, the risk of having NAFLD was similar in Asian and Caucasian (3-fold) and Hispanic individuals (4-fold) but was much higher in Black patients (9-fold) [110]. A considerable bulk of the literature supports genetic, metabolic, and dietary differences among African, Hispanic, and Asian individuals (reviewed in [111], though most studies were performed in the U.S.). In Hispanic individuals with American ancestry (Mexican-, Central, and South American), the *PNPLA3* rs738409 G allele frequency is higher than those from European or Afro-Caribbean backgrounds [112]. The rs738409 variant was found to be more common in Hispanic individuals (49%) compared to African American (17%) and European individuals (23%), corresponding to a high prevalence of NAFLD in Hispanic individuals and a low prevalence in African American individuals [100]. A case–control study performed on a cohort of obese Hispanic children with obesity in the U.S. showed that children with NAFLD had a higher percentage of the *PNPLA3* GG genotype at 70.2% versus 31.0% in non-NAFLD [113].

Transmembrane 6 superfamily 2 (*TM6SF2*) codes a lipid transporter with transmembrane domains in the endoplasmic reticulum and endoplasmic reticulum (ER)-Golgi intermediate compartment in the hepatocytes [98]. *TM6SF2* activity is required for normal very-low-density lipoprotein (VLDL) secretion, and its impaired function contributes to NAFLD [98]. The rs58542926 variant is a *Glu167Lys* missense mutation that alters serum lipid profiles in humans. As *TM6SF2* controls hepatic lipid efflux, decreased effects by deletions or mutations diminish lipoprotein secretion, causing triglyceride accumulation within the hepatocyte [114]. After the PNPLA3 I148M variant, the *TM6SF2* E167K (rs58542926) genetic variant has the most significant effect on NAFLD susceptibility [115]. The rs58542926 shows a 7% frequency in the MEC and is associated with a 35% increase in the risk of NAFLD [108]. Patients with the *TM6SF2* E167K variant have severe fatty liver disease, decreased apolipoprotein B100 levels, and lower circulating lipids [98]. The TM6SF2-T allele mutation E167K had similar low frequencies between Hispanic individuals (5%) [108,112] and those of European ancestry (8%) [108] and was strongly associated with ALT levels [88].

Membrane-Bound O-acyltransferase Domain Containing 7 (*MBOAT7*) gene encodes an enzyme involved in hepatic phospholipid remodelling by transferring polyunsaturated fatty acids to lysophospholipids [116]. Genetic studies indicate that a low level of *MBOAT7* in a human liver cell increases the severity of NAFLD and an inactivating mutation increases the risk of fat accumulating in the liver [116]. A polymorphism (rs641738) in the locus carrying the membrane-bound *MBOAT7* gene has been associated with the risk and severity of NAFLD through the suppression of *MBOAT7* at the messenger RNA and protein levels and altered phosphatidylinositol profiles [117]. It is associated with NAFLD development and the progress and severity of fibrosis and HCC in patients without cirrhosis [118]. In Europeans, a T mutation of the *MBOAT7* gene (rs641738) has been associated with NAFLD severity in those with TT homozygosity [117]. The study by Mansoor et al., 2021, on a cohort of obese Hispanic children in the U.S. showed that, while NAFLD was significantly increased with *PNPLA3* rs738409 C > G polymorphism ((OR = 3.7 (1.5–9.4)), the odds of NAFLD in the presence of the *MBOAT7* rs641738 variant was lower (OR = 0.19 (0.04–0.83)) [113]. Another study by Umano and colleagues (2018) has shown that another gene variant in the *MBOAT7* gene (rs626283) is associated with NAFLD and impaired insulin sensitivity in obese Caucasian children and adolescents but not among Hispanic and African American children and youths [119].

The glucokinase regulator (*GCKR*) gene codes a regulatory protein in the hepatocytes, negatively regulating glucokinase in response to fructose-1-phosphate and modulating glucose uptake in the liver [118]. The *GCKR* rs1260326 variant is a loss-of-function mutation that increases hepatic glucose uptake and malonyl-CoA concentration, providing more substrates for de novo lipogenesis. It shows a 37% frequency in the MEC and is associated with a 16% increase in the risk of NAFLD [108]. The variant has been linked to NAFLD development and fatty liver in obese youths [118]. Two single nucleotide polymorphisms in *GCKR* (rs1260326 and rs780094) are considerably related to NAFLD in African Americans and Latinos and were significantly associated with 16% (rs1260326) and 14% (rs780094) increases in NAFLD risk [108].

Besides what was discussed above, some genetic variants have now been recognized to protect individuals against NAFLD. A different allele of *PNPLA3* (rs6006460(T), encoding S453I) is associated with decreased hepatic triglyceride content [104]. Additionally, some splice variants in the gene encoding hydroxysteroid 17-beta dehydrogenase 13 (*HSD17B13*) resulting in loss of function, while not being associated with hepatic triglyceride content change, may protect against chronic liver damage [120,121]. Such differences in the genetic components not only can explain the heterogeneity associated with the diversity of susceptibility to NAFLD but also act as reliable susceptibility markers to help identify at-risk individuals at earlier ages. This could help better disease management through timely patient education, early interventions, and lifestyle changes.

In a recent study, Kubiliun and colleagues (2022) [122] undertook a case–control study on a multiethnic fatty liver disease cohort to assess the impact of known genetic variants, both individually and together, on racial/ethnic differences in the prevalence of fatty liver disease (of both NAFLD and alcoholic fatty liver disease etiologies). They calculated a weighted genetic risk score to combine the impact of known genetic variants (risk-increasing alleles included *PNPLA3* I148M (rs738409), *TM6SF2* E167K (rs58542926), *GCKR* P446L (rs1260326), and *TMC4/MBOAT7* (rs641738) and risk-decreasing alleles included *PNPLA3* S453I (rs6006460), *HSD17B13* A192fs (rs80182459), and *HSD17B13* splice (rs72613567) [122]). The study revealed that Black individuals generally carried fewer, whereas Hispanic individuals carried more risk-increasing alleles than White individuals. Differences in the frequency of risk alleles accounted for a large portion of the odds of fatty liver disease increase among Hispanic individuals compared to White individuals. Still, they failed to fully explain the lower odds of fatty liver disease in Black individuals, since after accounting for genetic risk score, age, sex, BMI and T2DM, the difference in the odds of FLD between Hispanic and White individuals became insignificant. Black individuals, however, remained at significantly lower odds of fatty liver disease than White individuals (OR = 0.21 (0.15–0.30)). This finding indicated that genetic protection might not be the only variable contributing to the protection of Black individuals against fatty liver [122].

Research performed outside the U.S. shows that polymorphisms in *PNPLA3* are frequent in Asian Indians with NAFLD, likely contributing to their increased prevalence of NAFLD [123]. A study by Bale et al., 2017, from India which comparing two distinct Indian ethnicities and showed that in South Indian ethnics, higher susceptibility to NAFLD was associated with the *TM6SF2* rs58542926 variant (OR = 2.7), while for North-East Indian ethnicities, an rs2281135 variant of *PNPLA3* gene was associated with higher susceptibility to NAFLD (OR = 2) [124]. A research study was recently published by Cavalcante et al., 2022, who employed a panel of 46 ancestry informative markers (AIM) to study the association between genetic ancestry with *PNPLA3* and *TM6SF2* polymorphisms in a group of adult Brazilians with biopsy-proven NAFLD [125]. An AIM is a single-nucleotide polymorphism which shows different frequencies between different ancestral populations [126]. In patients with simple fatty liver with European, Asian, or Native American ancestry, there was no significant difference between subjects with the *PNPLA3* G genotype and those with the CC genotype. However, in subjects with African/Black ancestry, the CC genotype was significantly more common than the G genotype (21.6% vs. 10.5%). Although no association was found between ancestry contribution and NASH, this finding still provides evidence in support of the favourability of African ancestry in the prognosis of NAFLD [126]. They, however, observed no association between ancestry contribution and the *TM62SF* genotypes in NAFLD [126].

Another interesting finding was reported by Zhang et al., 2014 [127], who compared the association of *PNPLA3* rs738409 polymorphisms with the risk of NAFLD among Han and Uyghur ethnics. While in both ethnicities, the *PNPLA3* GG allele but not the G.C. allele was in general associated with a higher risk of NAFLD (OR = 5.22 (1.94–14.04) in Han ethnics and 4.29 (1.60–11.48) in Uyghurs) compared to CC allele, the effect was sex-dependent. In Uyghurs, the association was significant only for males (males OR = 4.34 (1.78–10.60) vs. females OR = 1.16 (0.42–3.16)) while in Han ethnics, the association was only significant in females (OR in males = 1.12 (0.49–2.55) vs. OR in females = 7.21 (2.22–23.35)) [127].

Gene polymorphisms implicated in NAFLD are not limited to the liver’s regulators of lipids and carbohydrate metabolism. Dysfunction of the immune system is also believed to contribute to haptic injury, which follows the accumulation of fatty acids in the liver [128]. Toll-like receptor 4 (*TLR4*) is a pattern recognition receptor that acts as an endotoxin sensor within the innate immune system and has been implicated in the pathogenesis of NASH [128,129]. Several *TLR4* single nucleotide polymorphisms have been described that are differentially distributed worldwide [130]. These *TLR4* polymorphisms can alter innate immune responses [131]. Given the central role of innate immunity in the progression of NASH [132], *TLR4* genetic polymorphisms may contribute to the differential susceptibility of different ethnic populations to the progression of NASH.

The human studies on the association of *TLR4* polymorphism and NAFLD are currently limited. A case–control study from Turkey showed that the *Asp299Gly* allelic variant of the TLR4 receptor was protective against NAFLD [133]. However, a similar study from India showed no association between *TLR4 Asp299Gly* polymorphism with NAFLD [134]. These two publications may not apply to the general population due to the low sample size (n < 200 per group) [133,134]. Hence, demonstrating the role of *TLR4* polymorphisms in the ethnic/racial differences in susceptibility to NAFLD awaits future research.

## 6. Limitations of Race/Ethnicity Studies in NAFLD

NAFLD has a complex etiology and is caused by a combination of environmental and genetic factors. A common challenge with medical literature on race and ethnicity is that individuals’ race, ethnicity, and geographical origin have often been used synonymously to differentiate between populations. While U.S. studies tend to group participants as Latino, Black, Asian, White, or Indigenous, it is essential to acknowledge that these racial classifications are subjective or not clearly defined [135]. The traditional racial categories, which are based on skin colour and facial features, have never provided an accurate representation of human biological variation, and with the rise of genetics sciences, they are increasingly proven obsolete and without scientific basis [136]. Moreover, even if assuming different human races existed at some distant time, several hundreds of years of human travel, immigration, and admixture with other races make it difficult to assign a race category to any individual in the present era. The race and ethnicity categories recommended and used by the OMB and Census Bureau are defined and aimed for policy-making purposes and are based on self-identification, which may not be proper for use in medical research and the study of the biological basis for disease etiology [26,30,31]. Furthermore, most research projects that employ these categories leave people with multiple races/ethnicities out, further undermining the findings’ generalizability.

Just like race, ethnicity is also a social construct [137]. Although it might still lack a clear-cut definition, ethnicity attempts to identify a group of people through language, religion, collective history, national origin, or other cultural characteristics [110]. While race might imply biological differences among groups, ethnicity distinguishes people more culturally and socially [110,135]. There have been recommendations to the U.S. congress to abandon standard racial categories and focus the research funds on ethnic or cultural categories instead [26]. Nevertheless, racial diversity among ethnicities, or ethnicities living in diverse geographical areas, could result in uncertainties in the applicability of race- or ethnicity-focused research results [136,138]. Environmental and genetic factors differ considerably within these pooled ethnic groups, impacting study results and limiting inferences drawn based on ethnicity or race. The generalizability of described ethnic differences in disease prevalence, the progression of the disease, and the response to treatment is therefore limited by the many intrinsic geographic and socioeconomic disparities within ethnic groups. For example, a study by Fleischman et al., 2014, showed that in the U.S., the prevalence of NAFLD in Hispanic individuals of Dominican origin (16%) and Hispanic individuals of Puerto Rican origin (18%), was significantly lower than that of Hispanic individuals of Mexican origin (33%) [138]. This observation raises the question of whether the inferences regarding the high prevalence of NAFLD among Hispanic individuals in the U.S. have been made considering diversity within the pooled ethnicity [138].

Nonetheless, despite all said, race and ethnicity are considered important variables in epidemiologic research. They have potential utility in many contextual settings, ranging from studies aimed at elucidating disease etiology, to applications in clinical settings, to targeting specific groups for prevention and intervention on a public health scale. However, unlike other variables in medical research such as age and sex, race and ethnicity are subjective and, therefore, more difficult to conceive, measure, or generalize any interpretations to others. Hence, racial/ethnic differences need to be interpreted with caution.

## 7. Conclusions

NAFLD is a progressing global endemic with a significant burden on healthcare systems. Despite the needed caution in interpreting race/ethnic research results in NAFLD, these variables remain important in NAFLD research. Ethnic differences in disease susceptibility can be responsible, at least partly, for the heterogeneity observed in prevalence studies regarding NAFLD prevalence. Ethnicity differences can yield valuable information regarding biological and genetic differences among people with similar cultural, dietary, and geographical backgrounds. Besides, information regarding ethnicity is precious in helping resource allocations, health promotion, and health policies. So far, the research indicates that the racial/ethnic differences in susceptibility to NAFLD could be linked to differences in lifestyle, diet, metabolic comorbidity profile, and genetic background, among others.

Ethnic differences from different parts of the world are still relatively scarce and specific to a few countries. Since the ethnic differences depend on environmental factors, socioeconomic status, and existing national health policies, they may not be applicable beyond national borders.

## Data Availability

Not applicable.
